# Daily functioning and symptom factors contributing to attitudes toward antipsychotic treatment and treatment adherence in outpatients with schizophrenia spectrum disorders

**DOI:** 10.1186/s12888-021-03037-0

**Published:** 2021-01-13

**Authors:** J. Leijala, O. Kampman, J. Suvisaari, S. Eskelinen

**Affiliations:** 1 Department of Psychiatry, South Ostrobothnia Hospital District, Huhtalantie 53, 60220 Seinäjoki, Finland; 2 Faculty of Medicine and Health Technology, Tampere University, Tampere, Finland; 3 Department of Psychiatry, Pirkanmaa Hospital District, Tampere, Finland; 4 Finnish Institute for Health and Welfare, Mental Health Unit, Helsinki, Finland; 5 Psychiatry, University of Helsinki and Helsinki University Hospital, Helsinki, Finland; 6 Department of Public Health Solutions, Mental Health Unit, National Institute for Health and Welfare, Helsinki, Finland

**Keywords:** Adherence, Compliance, Psychotic disorder, Attitudes toward neuroleptic treatment scale, Clozapine

## Abstract

**Background:**

Poor adherence and negative attitudes to treatment are common clinical problems when treating psychotic disorders. This study investigated how schizophrenia core symptoms and daily functioning affect treatment adherence and attitudes toward antipsychotic medication and to compare patients using clozapine or other antipsychotics.

**Method:**

A cross-sectional study with data from 275 patients diagnosed with schizophrenia spectrum disorder. Patients adherence, attitudes, insight and side-effects were evaluated using the Attitudes toward Neuroleptic Treatment scale. Overall symptomology was measured using the Brief Psychiatric Rating Scale (BPRS), the Health of the Nation Outcome Scale (HoNOS). The functioning was assessed using activities of daily living scale, instrumental activities of daily living scale and social functioning of daily living scale.

**Results:**

Self-reported treatment adherence was high. Of the patients, 83% reported using at least 75% of the prescribed medication. Having more symptoms was related with more negative attitude towards treatment. There was a modest association with functioning and treatment adherence and attitude toward antipsychotic treatment. Attitudes affected on adherence in non-clozapine but not in clozapine groups.

**Conclusion:**

Early detection of non-adherence is difficult. Systematic evaluation of attitudes toward the treatment could be one way to assess this problem, along with optimized medication, prompt evaluation of side effects and flexible use of psychosocial treatments.

## Background

Schizophrenia is a chronic and often disabling illness, usually requiring maintenance treatment with antipsychotic medication. The majority of patients experience several relapses during course of the illness [1–3], whereas antipsychotic medication use reduces the risk of relapse markedly [4–8]. Rate of medication non-adherence in schizophrenia is reported to be 20–72% [9]. It has been estimated that as many as 60% of the patients stop medication use after 2–3 months, and 80% after 2 years [10]. Discontinuation of medication increases the relapse risk fivefold [11].

Poor adherence and negative attitudes to treatment are common clinical problems in psychiatry when treating psychotic disorders. Poor adherence to treatment is often noticed when the patient does not show up to an appointment or suffers a relapse. Although adherence, attitudes to treatment and insight are often assessed in a research setting, their systematic and structural evaluation in clinical practice is rare. Recent review found negative attitudes toward medication to be the primary reason directly associated with intentional non-adherence [12].

Patient adherence to medication is multifactorial and several patient related factors have been identified as reasons for non-adherence. Factors such as poor insight [13–18], substance abuse [14, 17, 19, 20], negative attitudes toward medication [9, 13, 17, 21, 22] and side-effects [19, 23] have been associated with non-adherence. Patients may also just forget to take their medicine, however better neurocognitive function does not automatic lead to better adherence [16, 24, 25].

Schizophrenia and other psychotic disorders often cause marked disability [26]. Individuals with schizophrenia and other non-affective psychotic disorders have significantly more problems in everyday functioning than the general population [27]. However, the role of everyday functioning in non-adherence has rarely been studied. There are only a few studies of associations between attitudes toward medication and psychopathology or functioning [28].

Clozapine is regarded as most effective antipsychotic medication to treat schizophrenia [29, 30] and the drug of choice for treatment resistance [31]. In Finland clozapine is widely used and it is routinely considered after failure of two different antipsychotic medications [32]. In a Finnish nationwide study the use of clozapine or long-acting depot injections was associated with lower risk of overall rehospitalization [33]. Time to discontinuation with clozapine has been longer than with other antipsychotics [34, 35]. In a 15-year naturalistic study the two most common causes for clozapine discontinuation were found to be non-adherence (35%) and side-effects (28%) [36]. Less is known about partial treatment adherence.

As it is so far unclear how functioning affect attitudes an adherence to antipsychotic medication, we investigated how attitudes and adherence are explained by psychiatric symptoms, daily functioning and compared adherence between patients using clozapine and those using other antipsychotic medications.

## Methods

### Subjects

In The Living Conditions and the Physical Health of Outpatients with Schizophrenia Study, a comprehensive clinical assessment and physical health examination was offered to all patients treated in the psychosis outpatient clinics in three municipalities from Southern Finland. This examination was voluntary, and patients were able to participate even if they refused the use of their information in the study. The recruitment procedure consisted of an invitation letter and a telephone call to each patient treated at the clinic. The data were collected between June 2009 and December 2013. The study was approved by the Ethics Committee of the Hospital District of Helsinki and Uusimaa and by the Hyvinkää Hospital Area. All participants gave a written informed consent.

### Instruments

Patient adherence, attitude, insight and side effects was measured using a self-report questionnaire Attitudes towards Neuroleptic Treatment scale (ANT) [37]. In the ANT attitude questions, the patients were asked to estimate in visual analogue scale from 0 to 100 the importance of medication in psychiatric treatment, readiness to take medications, how the medication influences one’s feelings, how the medication influences on one’s ability to think and how the medication will influence autonomy. The ANT attitude variable was formed from average of the five attitudes related ANT scale questions. In the ANT adherence question, the patients were asked to rate in five-point scale (0, 25, 50, 75 or 100%) how much they had used of prescribed psychiatric medications during the last 4 weeks. The ANT adherence was not normally distributed so dichotomous ANT adherence variable (0 (poor); 0–75%, 1 (good); > 75) was used in the final analysis. In the ANT side effect item, the patients were asked to estimate on visual analogue scale from 0 to 100 how much medication causes side effects. In the ANT insight variable, the patients were asked to estimate on visual analogue scale from 0 to 100 the extent of their current mental problems. In the ANT scales, low scores translate to negative attitude, poor insight, more side effects and poor adherence.

The patients’ current psychiatric state was assessed with the Health of the Nation Outcome Scale (HoNOS) [38], the 24-item version of the Brief Psychiatric Rating Scale (BPRS) [39], Global Assessment of Functioning (GAF) [40], and three global scores – alogia, anhedonia and apathy from the Scale for the Assessment of Negative Symptoms (SANS) [41]. The HoNOS was developed during the early 90s by the Royal College of Psychiatrists as a clinical measure of the health and social functioning of people with severe mental illness [38]. The HoNOS is divided into four subscales: behaviour, impairment, symptoms and social. Total score of HoNOS was used as the HoNOS total variable, and each subscale scores in HoNOS as behaviour, impairment, symptoms and social variables. The total sum score of 24-item BPRS was used for “BPRS total” variable and BPRS positive symptoms sum score from items measuring grandiosity, suspiciousness, hallucinations, unusual thought content, bizarre behaviour, disorientation and conceptual disorganization for the “BPRS positive symptoms” variable [42]. GAF score was used in the GAF variable and SANS item sum score in the SANS variable [40, 41]. All evaluations were performed by a trained research nurse.

Patients activities of daily living (ADL), instrumental activities of daily living (IADL) and social functioning were measured using self-report question based on measures developed by Katz et al., Lawton and Brody and the OECD [43–45]. In ADL, IADL question and social functioning questions patients were asked to rate in four-point scale (1 = no difficulties, 2 = some difficulties, 3 = much difficulties, 4 = unable to perform at all) how much difficulties they have in performing daily tasks. The questions were formulated as follows: “How do you nowadays manage the following activities?”. The ADL variable questions were getting in and out of bed, dressing, eating, bathing and toileting. The IADL variable questions were shopping, cooking, laundering, heavy cleaning and cutting toenails. The social functioning variable questions were using the phone, taking care of matters together with other people, communicating with strangers, dealing with the authorities and financial institutions. The questionnaire has been previously used in Finnish general population surveys [27].

Participants’ daily dose of antipsychotic medication was converted to chlorpromazine equivalents [46].

### Statistical methods

Statistical analyses were performed using SPSS software ver. 24.0 (IBM, Armonk, NY, USA). Demographic and clinical characteristics of the patient sample were calculated using descriptive statistic, and comparisons between non-clozapine and clozapine groups were done using the Mann-Whitney *U*-test. The exploration of the data was started by calculating the correlations between the response and the explanatory variables and grouping variables in meaningful factors. The upper limit of the correlation between the explanatory variables was set to 0.5 (Pearson correlation, r > 0.5) to avoid multicollinearity. One exception was made: the IADL and social functioning variables had correlation 0.64 but using both variables in the models is relevant to the research question. Variance inflation factor was calculated to test for multicollinearity in regression models, but no multicollinearities were found in any of the models. All VIF values were below 2.5.

A hierarchical linear regression model was used in analysing the effects of symptoms and daily functioning on ANT attitude score. The model was adjusted for age, gender, daily dose of antipsychotic medication, the use of clozapine, insight, experienced side effects and adherence. The model building was done using forward selection method to find best subset of variables. The final models (a, b, c, d) include psychopathology (BPRS total (a), BPRS positive symptoms (b), HoNOS total (c), HoNOS behaviour, HoNOS social, HoNOS impairment, HoNOS symptoms (d)) variables and the functional variables ADL, IADL and social functioning. The SANS and GAF variables were excluded from the final models. In this sample, 42% of the participants used clozapine. Because clozapine is not regarded as a first line treatment the clozapine users are likely to differ from other patients. Therefore, the data was split to clozapine and non-clozapine groups for a more detailed comparison. The adjusted R square was used to compare superiority of the models.

A hierarchical logistic regression model was used in analysing the effects of symptoms and daily functioning on the dichotomized ANT adherence score. The model was adjusted for age, gender, daily dose of antipsychotic medication, the use of clozapine, insight, experienced side effects and attitudes toward treatment. The final models (e, f, g, h) include psychopathology (BPRS total (e), BPRS positive symptoms (f), HoNOS total (g), HoNOS behaviour, HoNOS social, HoNOS impairment, HoNOS symptoms (h)) variables and the functional variables ADL, IADL and social functioning. The Nagelkerke R square was used to compare superiority of the models. The variables which were not statistically significant or relevant for comparison purposes were excluded from the final models.

## Results

Of the 409 patients who were sent an invitation, 275 completed the study protocol, the participation rate being 67.5%. The physical health findings of the sample have been reported by Eskelinen et al. [47].

Clinical characteristics of the study group are presented in Table 1. The mean age (± standard deviation) of the participants was 44.9 ± 12.6 years, and 152 (55.1%) were men. Of the participants, 68.8% had schizophrenia, 17.8% had schizoaffective disorder and 13.4% had other schizophrenia spectrum psychoses. Self-reported treatment adherence was high: 82,6% of patients reported using at least 75% of the prescribed medication. The mean ANT attitude score 72 (±16) corresponds to willingness to take medication, understanding the importance of medication for getting better, experiencing that the medication has a positive effect, helps in thinking and increases the sense of autonomy. Participants insight varied from poor to good with the mean ANT insight 50 (±25), and on the average participants recognized having some mental problems. The mean ANT side effect was 62 (±24) meaning that adverse effects were common but usually mild in severity. (Table 1).

**Table 1:** Demographic and clinical characteristics of the patient sample, and comparisons between non-clozapine and clozapine groups

	Total (*n* = 276)		Non-clozapine group (*n* = 161)		Clozapine group (*n* = 115)		*p*
	Mean or *n* (%)	S.D.	Mean or *n* (%)	S.D.	Mean or *n* (%)	S.D.	
Male sex *n* (%)	152 (55.1%)		85 (52.8%)		67 (58.3%)		0.368
Schizophrenia	190 (68.8%)		89 (55.3%)		101 (87.8%)		**< 0.001**
Schizoaffective disorder	49 (17.8%)		36 (22.4%)		13 (11.3%)		**0.018**
Other schizophrenia spectrum disorder	37 (13.4%)		36 (22.4%)		1 (0.9%)		**< 0.001**
Age	44.9	12.6	47.2	12.5	41.8	12.1	**0.001**
Chlorpromazine equivalents, mg	582.5	423.2	415.3	344.1	816.7	413.6	**< 0.001**
ANT-attitude *(0–100)*	72.0	16.5	71.1	17.0	73.3	15.8	0.334
ANT-adherence (over 75%)	228 (82.6%)		124 (77.0%)		104 (90.4%)		**0.004**
ANT-insight *(0–100)*	49.5	24.9	46.5	24.7	53.7	24.7	**0.024**
ANT-side effects *(0–100)*	62.4	24.0	64.0	25.5	60.2	21.6	0.175
BPRS (24–168)	34.6	7.8	34.4	8.1	34.9	7.4	0.363
BPRS positive symptoms *(7–49)*	10.2	4.3	10.1	4.4	10.3	4.1	0.58
HoNOS total *(0–48)*	7.2	4.5	7.1	4.5	7.3	4.4	0.712
HoNOS behaviour *(0–12)*	0.3	0.7	0.4	0.8	0.2	0.6	**0.02**
HoNOS impairment *(0–8)*	1.8	1.6	1.8	1.7	1.7	1.5	0.853
HoNOS symptoms *(0–12)*	3.0	2.2	2.9	2.2	3.3	2.2	0.139
HoNOS social *(0–16)*	1.2	1.3	1.2	1.4	1.2	1.3	0.591
SANS *(0–15)*	1.1	1.0	1.1	1.0	1.1	1.0	0.712
GAF *(0–100)*	56.1	12.6	57.6	12.5	54.0	12.5	**0.022**
ADL *(5–20)*	5.7	1.3	5.8	1.5	5.4	0.9	0.061
IADL *(5–20)*	7.6	2.8	7.8	3.1	7.3	2.3	0.422
Social functioning *(4–16)*	5.5	1.9	5.6	1.9	5.5	1.8	0.897

High score in ANT -attitude, −insight and -side effect indicates positive attitudes toward medication, better sickness awareness and less experienced side effects. High score in BPRS, BPRS positive symptoms, HoNOS total, HoNOS behaviour/impairment/symptoms/social and SANS indicates more severe symptom. High score in GAF indicates better functioning. High score in ADL, IADL and social functioning indicates more problems with activities of daily living

The patients using clozapine were younger and had higher medication dosage, better treatment adherence and insight. Schizophrenia was more common among clozapine users while schizoaffective disorder and other schizophrenia spectrum disorders were more common among those not using clozapine. The clozapine users had significantly lower GAF score than other patients but did not differ significantly in symptom measures (Table 1).

In linear regression analysis with the ANT attitude score as the outcome variable, BPRS total, BPRS positive symptoms, HoNOS total and HoNOS symptoms had a statistically significant effect on attitudes (Table 2). Increase in the BPRS or the HoNOS scores were associated with lower ANT attitude score, meaning that having more symptoms was related to more negative attitude towards treatment. In the HoNOS -scale the variation in attitudes was mainly explained by the HoNOS symptoms dimension. The ANT adverse effects, the ANT adherence and the daily dose of antipsychotic medication explained variations in all linear regression models. A lower number of side effects and a higher dose of antipsychotic medication were associated with a more positive attitude. The adjusted R square in this best fitting model was 0.20 (*p* < 0.001).

**Table 2:** Results of linear regression models explaining ANT –attitude scores in the total sample, clozapine and non-clozapine groups

	Model a	Model b	Model c	Model d
Total sample	Beta	Beta	Beta	Beta
Age	0.02	0.05	0.02	0.01
Sex (male)	0.07	0.05	0.07	0.06
Chlorpromazine equivalent	0.16*	0.18*	0.15*	0.17*
ANT-insight	0.14*	0.07	0.11	0.15*
ANT-side effects	0.30***	0.28***	0.28***	0.27***
ANT-adherence	0.17**	0.17**	0.17*	0.15*
BPRS_total	−0.27***			
BPRS_positive symptoms		− 0.25***		
HoNOS_total			−0.20**	
HoNOS_behaviour				−0.06
HoNOS_impairment				0.02
HoNOS_symptoms				−0.19*
HoNOS_social				−0.10
ADL	0.10	0.08	0.11	0.09
IADL	−0.03	−0.06	−0.05	− 0.07
social functioning	−0.04	− 0.03	−0.07	− 0.06
Adjusted R2	0.20	0.19	0.16	0.17
*p* for the model	< 0.001	< 0.001	< 0.001	< 0.001
**Non-clozapine group**				
Age	−0.07	−0.06	−0.07	− 0.07
Sex (male)	0.14	0.14	0.15	0.16
Chlorpromazine equivalent	0.19*	0.21**	0.18*	0.21*
ANT-insight	0.17	0.12	0.19*	0.24*
ANT-side effects	0.27**	0.27**	0.24**	0.22*
ANT-adherence	0.24**	0.23**	0.22*	0.21
BPRS_total	−0.19*			
BPRS_positive symptoms		−0.24**		
HoNOS_total			−0.20*	
HoNOS_behaviour				−0.09
HoNOS_impairment				−0.03
HoNOS_symptoms				−0.23*
HoNOS_social				−0.06
ADL	0.18	0.14	0.22*	0.19*
IADL	−0.03	−0.03	−0.08	− 0.09
Social functioning	−0.11	− 0.12	−0.15	− 0.12
Adjusted R2	0.23	0.25	0.20	0.22
*p* for the model	< 0.001	< 0.001	< 0.001	< 0.001
**Clozapine group**				
Age	0.14	0.17	0.13	0.14
Sex (male)	−0.07	−0.09	−0.07	− 0.10
Chlorpromazine equivalent	0.09	0.07	0.06	0.09
ANT-insight	0.22	0.08	0.14	0.11
ANT-side effects	0.35**	0.30**	0.37**	0.35**
ANT-adherence	0.10	0.11	0.14	0.10
BPRS_total	−0.41***			
BPRS_positive symptoms		−0.26*		
HoNOS_total			−0.26*	
HoNOS_behaviour				−0.05
HoNOS_impairment				0.11
HoNOS_symptoms				−0.09
HoNOS_social				−0.29*
ADL	−0.08	−0.02	− 0.07	−0.05
IADL	−0.04	−0.12	− 0.06	−0.09
Social functioning	0.00	0.07	0.03	0.08
Adjusted R2	0.22	0.12	0.15	0.13
*p* for the model	< 0.001	0.015	0.007	0.022

* *p* < 0.05 ** *p* < 0.01 ***p < 0.001

In the presentation of categorical variables: a positive estimate indicates a more positive attitude in women; a positive estimate indicates a more positive attitudes in the group with adherence level over 75%. High score in ANT -insight and -side effect indicates positive attitudes toward medication, better sickness awareness and less experienced side effects. All models adjusted by age, gender, daily antipsychotic dose, ANT-insight, ANT-adherence and ANT-adverse effects

The BPRS and the BPRS positive symptoms had statistically significant effects in both clozapine and non-clozapine groups when corresponding linear regression analyses were done separately. (Table 2). The most significant association (*p* < 0.001) with the BPRS total variable was found in the clozapine group. The HoNOS total score was a significantly explaining variable in clozapine group but not in non-clozapine group. Of the HoNOS subscales the HoNOS symptoms explained variation in attitudes (*p* < 0.05) in the non-clozapine and, while the HoNOS social explained variation (*p* < 0.05) in clozapine group. The chlorpromazine equivalent was explaining variable (*p* < 0.05) in non-clozapine group but not in clozapine group. The ANT adverse effects explained variation (*p* < 0.05) both groups, but ANT adherence only in non-clozapine group with the BPRS/BPRS positive symptoms/HoNOS total variables. The ADL variable explained variance (*p* < 0.05) in two models in the non-clozapine group. GAF and SANS -variables were not significant in any model and where therefore dropout for final models.

In logistic regression analysis of the total sample with the ANT adherence class as the outcome variable, the ANT attitude explained the variance (*p* < 0.05 – *p* < 0.01) in all models. Whereas BPRS total, BPRS positive symptoms, HoNOS total, HoNOS subscales and the SANS and GAF did not. (Table 3). In analysis of the total sample, a chlorpromazine equivalent was significant explainer of variation (*p* < 0.05) in one model. The best fitting model classified 83.9% of the cases correctly and had Nagelkerke R^2^ 0.25 (*p* < 0.001).

**Table 3:** Results of logistic regression analyses explaining ANT-adherence in the total sample, clozapine and non-clozapine groups

	Model e	Model f	Model h	Model g
**Total sample**	**OR**	**OR**	**OR**	**OR**
Age	1.01	1.02	1.02	1.01
Sex	2.17	1.99	1.63	2.14
Chlorpromazine equivalent	1.00	1.00	1.00	1.00*
ANT-attitude	1.04**	1.04**	1.03*	1.03*
ANT-insight	1.00	1.00	1.00	1.01
ANT-adverse effects	1.02	1.02	1.01	1.01
BPRS_total	0.97			
BPRS_positive symptoms		0.95		
HONOS_total			0.96	
HONOS_behaviour				1.24
HONOS_impairment				0.94
HONOS_symptoms				0.75
HONOS_social				1.20
ADL	0.73	0.73	0.77	0.74
IADL	1.19	1.22	1.2	1.25
social functioning	0.82	0.84	0.80	0.80
Nagelkerke R square	0.22	0.23	0.21	0.25
*p* for the model	< 0.001	< 0.001	< 0.001	< 0.001
**Non-clozapine group**				
Age	1.03	1.04	1.04	1.04
Sex	1.67	1.46	1.15	1.64
Chlorpromazine equivalent	1.00	1.00	1.00	1.00
ANT-attitude	1.05**	1.05**	1.04*	1.04*
ANT-insight	1.00	1.00	0.99	1.00
ANT-adverse effects	1.02	1.02	1.01	1.01
BPRS_total	0.98			
BPRS_positive symptoms		0.98		
HONOS_total			1.03	
HONOS_behaviour				1.45
HONOS_impairment				1.06
HONOS_symptoms				0.79
HONOS_social				1.34
ADL	0.76	0.73	0.79	0.74
IADL	1.07	1.16	1.11	1.11
social functioning	0.85	0.85	0.79	0.84
Nagelkerke R square	0.29	0.30	0.26	0.33
*p* for the model	< 0.01	< 0.001	< 0.01	< 0.01
**Clozapine group**				
Age	0.99	1.00	0.99	0.93
Sex	3.82	4.23	4.23	19.31
Chlorpromazine equivalent	1.00	1.00	1.00	1.00
ANT-attitude	1.04	1.04	1.05	1.02
ANT-insight	1.00	1.00	0.98	1.00
ANT-adverse effects	1.02	1.03	1.02	1.07
BPRS_total	0.99			
BPRS_positive symptoms		0.96		
HONOS_total			0.93	
HONOS_behaviour				14.71
HONOS_impairment				0.39
HONOS_symptoms				0.78
HONOS_social				1.11
ADL	2.50	2.66	1.92	2.82
IADL	1.58	1.50	1.55	2.37*
social functioning	0.78	0.84	0.79	0.71
Nagelkerke R square	0.24	0.25	0.30	0.38
*p* for the model	0.31	0.28	0.21	0.15

* *p* < 0.05 ** *p* < 0.01 ***p < 0.001

In the presentation of categorical variables: a positive estimate indicates a better adherence for women. A positive estimate indicates a better adherence related to higher score in ANT-scales. High score in ANT -attitude, −insight and -side effect indicates positive attitudes toward medication, better sickness awareness and less experienced side effects. All models adjusted by age, gender, daily antipsychotic dose, ANT-insight, ANT-attitude and ANT-adverse effects

The non-clozapine and the clozapine groups differed from each other (Table 3). The ANT attitude explained the variance (*p* < 0.05 – *p* < 0.01) in non-clozapine group but not in clozapine group. The IADL variable was significant explainer of variation in one model in clozapine group. The higher IADL score increased risk for good adherence. The ADL, IADL and the social functioning did not explain variation in adherence class on a statistically significant level in other models.

## Discussion

The main aim of this study was to explore how attitudes toward antipsychotic treatment and treatment adherence are explained by symptoms and everyday functioning. Having less severe symptoms predicted more positive attitude towards antipsychotic treatment, while also having a higher dose and less side effects also predicted more positive attitude. We used more comprehensive ADL, IADL and social functioning -scales instead of GAF to analyse if daily functioning affect adherence and attitude toward antipsychotic medication. Lower scores on GAF have been reported to associate with poor adherence [28, 48]. There was a modest connection between everyday functioning and attitudes towards antipsychotic treatment. The overall self-reported adherence was good and even better with clozapine users. Better treatment adherence in patients using clozapine is consistent with previous register-based studies [49]. We found that overall symptoms did not explain non-adherence. Poor adherence was related to more negative attitudes toward antipsychotic medication in non-clozapine group. Whereas in one model in clozapine group functioning limitations measured as IADL explained variation in adherence.

Higher scores in overall psychopathology were mostly connected with negative attitudes to antipsychotic medication. This finding is in line with previous studies of attitudes toward antipsychotic medication in schizophrenia [28]. Assessing attitude with a specific measure, such as the BPRS, produces the highest explanatory level. Both the BPRS and the HoNOS explained markedly the medication related attitudes. The HoNOS provided a similar result as the broader BPRS. This may reflect the fact that psychopathology is an essential part of the HoNOS. Corresponding separate analyses with clozapine and non-clozapine subgroups showed somewhat different results. In the non-clozapine group, the HoNOS symptoms was a significant explaining factor, whereas in the clozapine group it was the HoNOS social subscale. The HoNOS social subscale includes items related to everyday functioning (problems with activities of daily living, problems with living conditions, problems with occupation and activities). Hence, a decrease in functional capacity in these daily affairs could lead to more negative attitudes towards medications among clozapine users. Instead in non-clozapine group the higher rate of ADL problems associated with more positive attitudes. It is possible that patients with more function impairment received social support which could reflect as more positive attitudes toward medication. Having higher dose of antipsychotic medication and less side effects was linked to more positive attitudes in patients treated with other antipsychotics than clozapine. It is quite probable that effective and well tolerated medication may lead to better attitudes toward treatment. Of note, having more severe symptoms was associated with more negative attitudes to the treatment, even after adjusting for the effect of insight, suggesting that this association could also be an understandable reaction to an inefficient treatment.

In this study the adherence rates were high and 228 (82.6%) of the participants were considered adherent with cut-off 75% usage of prescribed medication. The adherence this high is suspect for bias, although high adherence rates are not totally uncommon finding [50]. A recent study using electronic adherence monitoring report 72.0% adherence to clozapine compared our finding of 90.4% adherence [51]. A selection bias with clozapine is one possible explainer of this finding; patients with poor adherence are unable to use clozapine and on other hand effective medication can lead to better attitude. The clozapine users in this study had a more severe disorder and had more functioning problems than people treated with other antipsychotics. The overall psychopathology measured with the BPRS and the HoNOS did not explain adherence measured with the ANT. This is in line with previous findings [52–54]. However, psychotic symptoms have also been linked to non-adherence [55, 56]. Different methods for assessing adherence to treatment can explain this contradiction between the results. The more negative attitudes toward antipsychotic medication where linked to poor adherence with patients using antipsychotic medication other than clozapine. This finding was not seen in clozapine group. We used ADL, IADL and social functioning -scales to evaluate if treatment adherence could be explained by functional limitations. In this study, we wound that functional capacity had no particular effect on adherence to treatment, except in one model, where function deficiencies were associated with better treatment adherence. Most likely patients with severe functional limitations received more social support, which can improve adherence to medication.

We compared two widely used scales, the HoNOS and the BPRS in association to adherence. The BPRS is considered as a specific scale for rating schizophrenia symptoms whereas the HoNOS is more universal. In the HoNOS the interviewer is asked to rate symptoms and evaluate whether immediate clinical action is needed, whereas in the BPRS rating the symptoms are the main questions to evaluate. This can lead to people with severe symptoms but good insight having lesser scores in the HoNOS than in the BPRS. The HoNOS symptoms subscale also catches other mental or behavioural problems. The best model showed good sensitivity for detecting adherent patients but failed to specify non-adherent. This is a common problem with adherence studies [57, 58]. The better treatment adherence with clozapine users can be explained by the patient selection bias, the better antipsychotic effect of clozapine and closer monitoring related to the medication.

The present study has several limitations. A cross-sectional study cannot support causal conclusions. In addition, patients who decline from treatment are probably less likely to participate in a study. Participating in a study may also improve adherence. Moreover, patients self-rated evaluations for everyday function, attitudes and adherence. Self-report scales are subject to bias and may overestimate adherence. The study subjects were long-term patients with schizophrenia, and it is likely that many of them had some cognitive deficits. In addition, self-rating everyday functioning by ADL/IADL can lead to underestimation of problems in daily living. The significance levels are unadjusted for multiple comparison causing potential issue of spurious findings.

## Conclusions

Adherence is a complex phenomenon and detecting non-adherence prior to hospitalization is difficult. When patients are treated with polypharmacy is an assessment of adherence even more difficult. The symptom and adherence monitoring should be made routinely using structural evaluations even when chronic illness is considered stable. Monitoring of medication related attitudes may help evaluating possible non-adherence. The negative attitudes are a potential target for therapeutic interventions and personalized psychoeducation. Assertive treatment of psychotic symptoms with effective medication, and screening and providing treatment to side-effects, could affect positively the medication related attitudes and adherence. The use of long acting depot injection can be beneficial because it is difficult to maintain an excellent or at least a good adherence to medication in real life over the years [50, 59].

## Data Availability

In research collaboration, sharing of the data is possible if the intended collaboration is concordant with the informed consent given by the participants and the general data protection regulation. It requires a separate agreement with the Hospital District of Helsinki and Uusimaa.

## Abbreviations

ADL: Activities of daily living
ANT: Attitudes towards Neuroleptic Treatment scale
BPRS: Brief Psychiatric Rating Scale
GAF: Global Assessment of Functioning
HoNOS: Health of the Nation Outcome Scale
IADL: Instrumental activities of daily living
OECD: The Organisation for Economic Co-operation and Development
SANS: Scale for the Assessment of Negative Symptoms
SPSS: Statistical Package for Social Sciences
VIF: Variance inflation factor
